# Microevolution of Group A Streptococci In Vivo: Capturing Regulatory Networks Engaged in Sociomicrobiology, Niche Adaptation, and Hypervirulence

**DOI:** 10.1371/journal.pone.0009798

**Published:** 2010-04-14

**Authors:** Ramy K. Aziz, Rita Kansal, Bruce J. Aronow, William L. Taylor, Sarah L. Rowe, Michael Kubal, Gursharan S. Chhatwal, Mark J. Walker, Malak Kotb

**Affiliations:** 1 Research Services, Veterans Affairs Medical Center, Memphis, Tennessee, United States of America; 2 Research Services, Veterans Affairs Medical Center, Cincinnati, Ohio, United States of America; 3 Department of Microbiology and Immunology, Faculty of Pharmacy, Cairo University, Cairo, Egypt; 4 Computation Institute, University of Chicago, Chicago, Illinois, United States of America; 5 Biomedical Informatics, Children's Hospital Medical Center, Cincinnati, Ohio, United States of America; 6 Health Science Center, University of Tennessee, Memphis, Tennessee, United States of America; 7 Helmholtz Centre for Infection Research, Braunschweig, Germany; 8 School of Biological Sciences, University of Wollongong, Wollongong, New South Wales, Australia; 9 College of Medicine, University of Cincinnati, Cincinnati, Ohio, United States of America; University of Hyderabad, India

## Abstract

The onset of infection and the switch from primary to secondary niches are dramatic environmental changes that not only alter bacterial transcriptional programs, but also perturb their sociomicrobiology, often driving minor subpopulations with mutant phenotypes to prevail in specific niches. Having previously reported that M1T1 *Streptococcus pyogenes* become hypervirulent in mice due to selection of mutants in the *covRS* regulatory genes, we set out to dissect the impact of these mutations in vitro and in vivo from the impact of other adaptive events. Using a murine subcutaneous chamber model to sample the bacteria prior to selection or expansion of mutants, we compared gene expression dynamics of wild type (WT) and previously isolated animal-passaged (AP) *covS* mutant bacteria both in vitro and in vivo, and we found extensive transcriptional alterations of pathoadaptive and metabolic gene sets associated with invasion, immune evasion, tissue-dissemination, and metabolic reprogramming. In contrast to the virulence-associated differences between WT and AP bacteria, Phenotype Microarray analysis showed minor in vitro phenotypic differences between the two isogenic variants. Additionally, our results reflect that WT bacteria's rapid host-adaptive transcriptional reprogramming was not sufficient for their survival, and they were outnumbered by hypervirulent *covS* mutants with SpeB^−^/Sda^high^ phenotype, which survived up to 14 days in mice chambers. Our findings demonstrate the engagement of unique regulatory modules in niche adaptation, implicate a critical role for bacterial genetic heterogeneity that surpasses transcriptional in vivo adaptation, and portray the dynamics underlying the selection of hypervirulent *covS* mutants over their parental WT cells.

## Introduction

Group A streptococci (GAS) are human pathogens that infect over 700 million children and adults each year [1]. Whereas the overall mortality rate of GAS infections is less than 0.1%, the mortality rate of invasive GAS infections, which have resurged in the past 30 years, mounts to 25% (out of >650,000 new cases per year) [1]. Among the various GAS serotypes, the globally disseminated M1T1 clonal strain remains the most frequently isolated from cases of invasive and non-invasive infections [2], [3], and although disease severity partially depends on host genetic factors [4], [5], [6], M1T1 GAS possesses unique genomic features that contribute to its evolutionary fitness [7], [8], [9], [10]. Among these features is the ability of M1T1 bacteria to switch to a hypervirulent phenotype associated with invasive diseases in vivo [11], [12], [13], [14], a phenomenon that is not fully understood and whose specificity to the M1 serotype remains to be established [15].

We previously reported that virulent representatives of M1T1 GAS, with the phenotype SpeB^hi^/SpeA^−^/Sda1^low^, irreversibly switch to the hypervirulent SpeB^−^/SpeA^+^/Sda1^high^ phenotype after ≥3 days in vivo [12], [16] and that the parent phenotype vanishes by day 7 post-infection [16]. Subsequent studies uncovered that this genetic switch is driven by host innate immune pressure that selects for bacteria with pathoadaptive mutations in the *covRS* genetic locus [13], [14]. CovRS is a two-component regulatory system, in which CovS transduces external signals [17], [18] to CovR, which in turn represses the transcription of several group A streptococcal (GAS) virulence gene sets, including the capsule synthesis operon (*hasABC*), the streptolysin S operon (SLS or *sagA-H*), and the streptokinase gene (*ska*) [19], [20]. CovR regulatory activity is thought to be triggered when the protein is phosphorylated at D53 [21], possibly by acetyl phosphate [22], [23]. On the other hand, under stress conditions, CovS dephosphorylates CovR and reverses virulence gene repression [21], [22].

Mutations in *covS* are thus expected to affect CovR phosphorylation status differentially in vitro and in vivo (under stress conditions), and to consequently modulate CovRS signaling-regulation circuits in a complex manner that remains largely unexplored. This complexity is further magnified by the reported strain-specific differences in the impact of CovS on pathogenesis [24], by the finding that phosphorylated CovR has different affinities to different streptococcal promoters [21], and by the possibility that CovR promoter binding may be modulated by kinases or phosphatases other than CovS [25]. In fact, CovR retains some of its functions in the absence of wild type CovS [26], and different *covS* mutations, albeit clustered in its histidine kinase domain, might have different effects on expression of CovR-regulated genes [27]. In accordance with these biochemical findings, we and others have reported that some *covS* mutations generate hypervirulent isolates associated with invasive forms of streptococcal infection [13],[14],[26],[28]. One of the most striking outcomes of these mutations is the constitutive repression of a gene encoding the key GAS cysteine protease, SpeB, which remodels the host-pathogen interface [29] by differentially degrading bacterial surface and secreted proteins [12], [30], [31] as well as host proteins [32], [33], [34]. Consequently, absence of a proteolytically active SpeB preserves several virulence factors that it normally degrades [12], [35]. One of these preserved factors is the highly potent DNase, Sda1, which destroys neutrophil extracellular traps, NETs [36], protecting the bacteria from neutrophil killing, promoting bacterial invasion, and facilitating human plasminogen-mediated bacterial dissemination into normally sterile sites, which results in invasive infections [14].

Despite the association between *covS* mutations and the emergence of the hypervirulent phenotype of M1T1 strains, it is unclear whether this increased virulence can be entirely attributed to the modulation of the CovR regulon or if other networks are also perturbed in vivo directly, indirectly, or independently of the CovRS system. Additionally, the effects of *covS* mutations on bacterial niche adaptability are still undetermined because the in vivo transcriptomes of the wild type (WT) and animal-passaged (AP) bacteria have not been compared under *the same* experimental conditions.

To address these issues and improve our understanding of the gene regulatory impact of mutational and adaptive events contributing to the hypervirulent phenotype of M1T1 strains, we analyzed differences in growth requirements, transcriptome profiles, and regulatory circuits of the virulent (WT) and hypervirulent (AP) phenotypes of the M1T1 strain both in vitro and during initial in vivo infection. Such comprehensive analyses highlighted the behavior of genomic subsystems that may be involved directly or indirectly in *S. pyogenes* niche adaptation and pathogenesis. In addition, this approach offered a rare transcriptional snapshot of the SpeB^hi^/SpeA^−^/Sda1^low^ population prior to its extinction in vivo.

## Results

### Phenotype Microarrays show no major nutritional or metabolic differences between wild type and animal-passaged, hypervirulent M1T1 GAS

We used Biolog**®** Phenotype Microarrays (PM) to screen 1900 different growth conditions, including a large set of different carbon and nitrogen sources, pH values and salt concentrations, as well as different concentrations of various antimicrobial agents (Fig. S1), and found that both WT and AP GAS have similar growth requirements in vitro with minor differences. For example, AP bacteria grew better than WT bacteria in the presence of N-acetyl-neuraminic acid, and were more sensitive to four antimicrobials (out of 373 screened in PM), including the calcium-specific metal chelator EGTA, the lipophilic chelator 5-chloro-7-iodo-8-hydroxyquinoline, and the antibiotics tobramycin and cefotaxime (Table 1). However, when both bacterial variants were grown at 37°C in the enriched Todd Hewitt broth medium, their growth rates were indistinguishable, and the only detectable difference was that AP bacteria were more buoyant in liquid culture compared to WT. This difference in buoyancy can be attributed to differences in proteolytic activity (WT >> AP) that may degrade many surface proteins, including pilin, or induce changes in the surface charge making bacterial aggregates more compact. The difference in buoyancy can also be attributed to differences in the expression of hyaluronic acid capsule (AP > WT).

**Table 1:** PM array differences.

PM code	PM Phenotype	AP relative to WT	
(Plate: well)		PM Score	Comment
PM02A: B02	N-acetyl-neuraminic acid utilization as carbon source	+88	Gain (Upregulation)
PM14A: H04	Sensitivity to EGTA (Ca^++^ chelator)	−74	Loss (Downregulation)
PM16A: A09, A10	Sensitivity to 5-chloro-7-iodo-8-hydroxyquinoline (lipophilic chelator)	−101	Loss (Downregulation)
PM12B: F04	Sensitivity to tobramycin (an aminoglycoside acting on protein synthesis)	−78	Loss (Downregulation)
PM16A: A01	Sensitivity to cefotaxime (a cephalosporin acting on cell wall)	−93	Loss (Downregulation)

Phenotypes gained or lost by the animal-passaged (AP) mutant strain as determined by the consensus of two independent runs of Phenotype Microarrays. PM score  =  a differential value reflecting the growth rate of AP relative to WT in minimal culture media containing different nutrients, chemicals, or antimicrobials.

Although the PM results confirm previous experiments [16] showing a few minor differences between WT and AP bacteria in vitro, this technology is limited because it uses minimal media and measures microbial respiration as a growth indicator (http://www.biolog.com). Minimal media are not optimal for the expression of GAS proteins, which may explain why GAS failed to grow at many of the PM conditions (Fig. S1) and why fewer phenotypic differences were observed between WT and AP bacteria than transcriptomic differences (see below).

### In vivo murine chamber infection model allows the dissection of regulatory vs. mutation-selection events

Several studies, including ours, have examined phenotypic, proteomic, and transcriptional differences between WT and animal-passaged M1T1 GAS strains [For example, 11,12,13,16,26,27,37], but none attempted to capture the dynamic changes in the bacterial population in vivo. Because bacteria recovered from animals and cultured in the laboratory are likely to have reprogrammed many regulatory networks to re-adapt to the in vitro growth conditions, transcriptome profiling of these bacteria may not reflect their actual in vivo gene expression and regulation.

**Figure 1:**
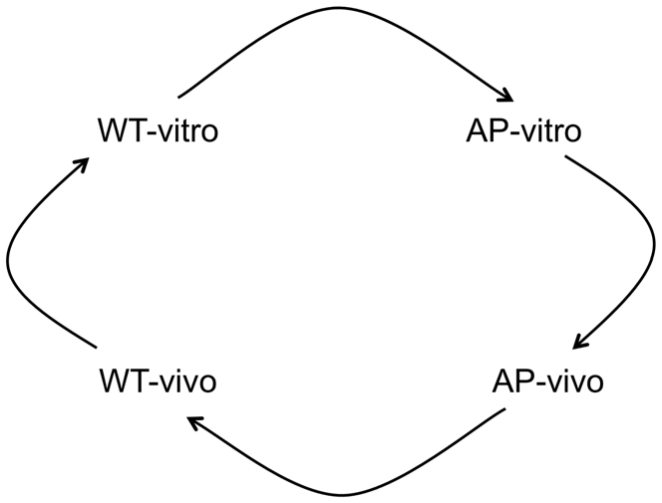
Hybridization scheme. Diagram showing the cyclic hybridization scheme followed in the microarray experiments.

In this study, we designed and performed in vivo passage experiments in which equal loads of in vitro-grown WT and AP bacteria were separately inoculated into murine subcutaneous chambers [16] and collected by needle aspiration after 24 h, a time sufficient for the bacteria to sense the host environment and transcriptionally respond to it, but not long enough to allow detectable restructuring of the bacterial community and selection of mutants. The concentrations of viable WT and AP bacteria at 24 h post-inoculation remained essentially the same: 1−5×10^9^ colony-forming units (CFU)/ml vs. ∼2×10^9^ CFU/ml inoculum. We extracted RNA from these bacteria immediately after their recovery from mouse chambers with no additional culturing and used the extracted RNA for transcriptome profiling as detailed in Materials and Methods. For transcriptional profiling, we followed a cyclic, two-color design and performed 28 oligonucleotide microarray hybridization experiments representing technical and biological replicates of the four cell states under investigation: WT grown in vitro (WT-vitro), WT grown in vivo (WT-vivo), AP grown in vitro (AP-vitro), and AP grown in vivo (AP-vivo), Fig. 1.

### Multiple approaches to microarray analysis show statistically significant and biologically relevant differences between the WT and AP populations in vitro and in vivo

To gain biologically relevant knowledge from the transcriptome studies without compromising statistical significance, we interrogated the data using multiple strategies that had been developed for microarray analysis and visualization, taking into consideration the strengths and limitations of each strategy.

**Figure 2:**
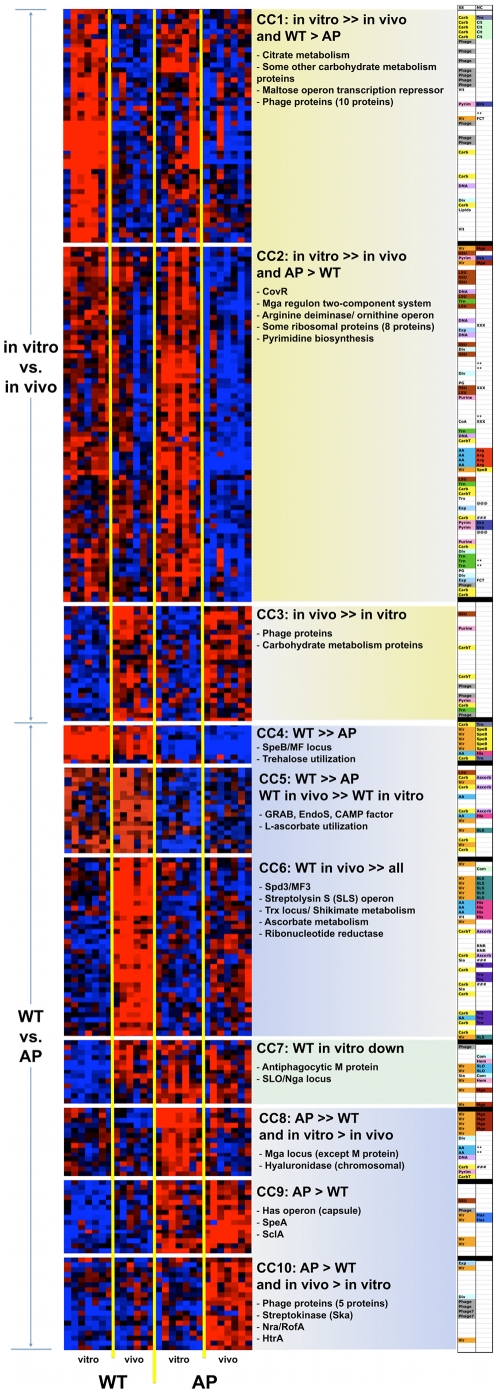
Pathovivogram of expression microarrays. Heat maps of clustered normalized expression values from biological replicates showing ten major coexpression clusters (CCs). Shades of red: upregulation; shades of blue: downregulation; black: expression value below threshold. CC1-CC3 are clusters that differentiate bacteria grown in vitro from those grown in vivo (vivogram) and represent the adaptational transcriptional program. CC4-CC10 are clusters that differentiate WT from AP bacteria (pathogram), all of which but CC7 represent transcriptional differences driven largely by the AP mutation. CC7 represents transcriptional differences driven *both* by mutation and by in vivo adaptation. The right column displays the subsystems (SS) and neighbor clusters (NC) to which these genes belong. A higher resolution version of this figure is provided online as Figure S2. Detailed annotations are provided in Table S1.

By clustering normalized expression values from different biological replicates in all data sets, we generated an overall “pathovivogram” that includes 276 genes in ten **c**oexpression **c**lusters, CCs (Fig. 2, Fig. S2, and Table S1). This pathovivogram highlights the transcriptional patterns that distinguish WT from AP bacteria regardless of their growth habitat (pathogram, Fig 2. CC4-CC6 and CC8-CC10), and the transcriptional patterns that are shared by WT-vitro and AP-vitro bacteria but that differentiate them from their corresponding in vivo samples (vivogram, Fig. 2, CC1-CC3). Moreover, we identified a unique cluster, CC7, that includes genes upregulated both in vivo and as a consequence of the *covS* mutation (e.g., those encoding M protein, streptolysin O, and nicotinamide adenine dinucleotide glycohydrolase (NADGH), Fig. 2, CC7).

The patterns in clusters CC1-CC3 describe those gene sets whose transcription was turned on (CC1-CC2) or off (CC3) to drive the bacterial adaptation to the host environment, but are not primarily related to bacterial pathogenesis. Many of these genes encode metabolic enzymes (e.g., carbohydrate metabolism, arginine degradation, and pyrimidine biosynthesis), ribosomal proteins (mostly downregulated), and sugar or peptide transport systems, reflecting the transition from a carbohydrate-rich laboratory medium to a protein-rich, oxygen-poor subcutaneous tissue. Such transition is bound to have major downstream effects on the bacterial virulence gene expression [38], [39], [40].

While the expression patterns in clusters CC4, CC5, CC8, and CC9 match the previously described in vitro differences between WT and AP strains [10], [12], [13], [26], [27] (e.g., downregulation of SpeB, CAMP, and EndoS genes; upregulation of SpeA, SIC, and capsule genes), the combined comparative analysis of in vitro and in vivo samples revealed novel expression patterns exemplified by CC6 and CC10. These two clusters include genes whose expression has been repressed (CC6) or induced (CC10) by the AP mutation, but the repression or induction becomes observable only in vivo. For example, the expression of genes in the SLS and Trx operons is induced in vivo only in WT bacteria, but is mostly repressed in AP bacteria (Fig. 2, CC6) both in vitro and in vivo. Likewise, some genes are only induced in AP-vivo, including those encoding the toxin/enzyme streptokinase and a RofA/Nra-like transcriptional regulator (Fig. 2, CC10).

Subsequent to the coexpression analysis, we calculated differential expression ratios between each pair of conditions (Table S2) at different statistical significance cutoffs, starting by the commonly used significance threshold of twofold ratio and *P* value <0.05, and moving to thresholds that are more conservative. At all significance cutoffs, the fewest observed transcriptional changes were those differentiating WT and AP bacteria in vitro compared to other pairs of conditions. By contrast, the most dramatic transcriptional reprogramming was that exhibited by the WT bacteria in their attempt to adapt to the in vivo environment (Table 2). Overall, at the least conservative statistical threshold (P<0.05), the transcription of 557 (23.9%) out of 2,329 genes probed in the microarrays was significantly perturbed (up or down, at one or more conditions, Fig. S3) and the only set of contiguous genes that underwent transcriptional changes at *all* tested conditions is the SpeB operon gene set (CC4 in Fig. 2). However, the fold-change in SpeB operon transcription level largely varied across the four experimental conditions, ranging from a 2.2-fold to a 30-fold downregulation (Table 3).

**Table 2:** Number and percentage of genes significantly different between each pair of conditions at different significance thresholds.

Statistical threshold	WT vs. AP in vitro	WT vs. AP in vivo	WT vitro vs. vivo	AP vitro vs. vivo
P<0.05	137 (5.9%)	167 (7.2%)	266 (11.4%)	203 (8.7%)
P<0.01	57 (2.45%)	59 (2.53%)	124 (5.32%)	95 (4.08%)
P<0.05 + FDR*	9 (0.39%)	9 (0.39%)	32 (1.37%)	20 (0.86%)

*Benjamini and Hochberg false-discovery rate (FDR) test.

**Table 3:** Expression ratios of selected virulence genes and regulators^1^.

Gene product	SF370 genome	M1T1 genome	AP/WT vitro	AP/WT vivo	WT vivo/vitro	AP vivo/vitro
HasA	SPy2200	+	6.24	6.70	*	*
NADGH	SPy0165	+	*	*	2.32	*
SLO	SPy0167	+	8.92	*	6.78	*
GAPDH	SPy0274	+	*	*	*	*
SpyA	SPy0428	+	*	*	*	*
Hypothetical protein	SPy0430	+	5.98	6.20	*	*
SLS	SPy0738	+	*	−4.35	3.00	*
IdeS/Mac	SPy0861	+	*	*	*	*
Hyl	SPy1032	+	*	*	*	*
CAMP	SPy1273	+	*	−2.25	*	*
DltA2	SPy1310	+	*	*	*	*
DltA1	SPy1312	+	*	*	*	*
GRAB	SPy1357	+	−2.44	−2.49	*	*
EndoS	SPy1813	+	−5.92	*	*	2.73
Ska	SPy1979	+	*	2.47	2.51	*
SclA	SPy1983	+	4.50	7.33	*	*
Fibronectin-binding	SPy2009	+	2.90	*	*	−3.24
C5a peptidase	SPy2010	+	3.32	*	*	−3.15
SIC	SPy2016	+	11.28	4.93	*	−3.69
M1 protein	SPy2018	+	4.08	*	2.15	*
SpeB	SPy2039	+	−13.16	−29.85	−2.29	−5.18
Spa^2^	−	−	*	3.27	*	*
**Superantigens**						
SmeZ	SPy1998	+	*	*	*	*
SpeA	−	+	2.37	4.50	*	3.54
SpeC	SPy0711	−	*	*	*	*
SpeG	SPy0212	+	*	*	*	*
SpeH^2^	SPy1008	−	*	*	−4.52	*
SpeI	SPy1007	−	*	*	*	*
SpeJ	SPy0436	+	*	*	*	*
**Streptodornases**						
Spd1/MF	SPy2043	+	−6.21	−7.87	*	−2.41
Spd2/MF2	SPy0712	−	*	*	*	*
Spd3/MF3	SPy1436	+	*	−3.01	2.73	*
Sda1^3^	−	+	5.66	*	6.51	*
**Regulators**						
RofA	SPy0124	+	*	*	*	*
RopA	SPy2037	+	*	*	−4.65	−4.46
RopB/Rgg	SPy2042	+	*	*	*	*
RALP3	SPy0735	+	*	3.26	−3.01	*
Nra (SPyM3_0097)^2^	?	?	*	3.43	*	*
TrxR	SPy1587	+	*	*	5.45	*
Mga	SPy2019	+	*	*	*	*

* The transcript was either not significantly altered, or its level was below detection threshold.

1 Values in the table are positive or negative fold-change ratios.

2 Although these genes are absent in M1T1, their probes cross-hybridized with M1T1 RNA.

3 There was no *sda1*-specific probe in the microarrays; the values shown here are qPCR data [14].

**Figure 3:**
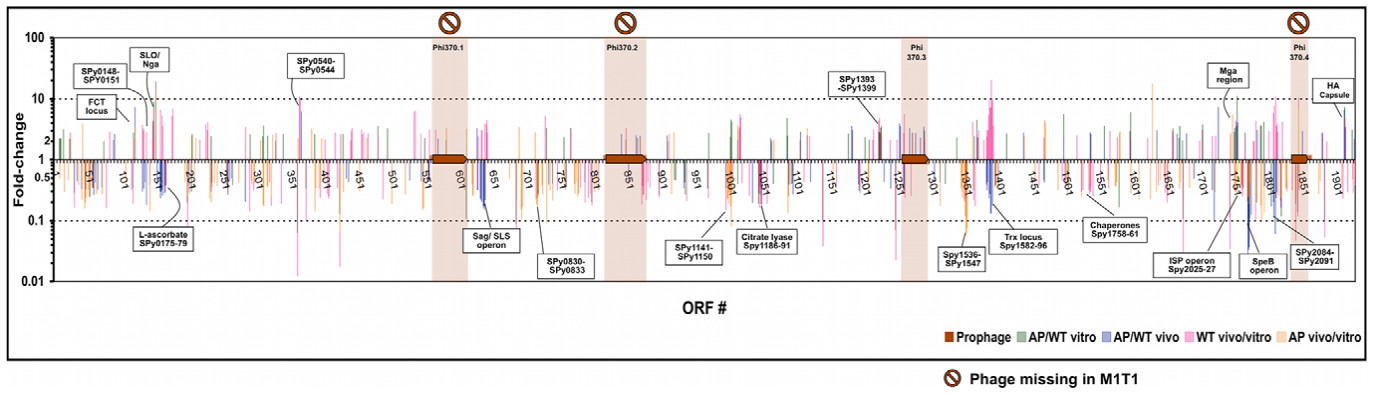
Neighbor clustering of significantly differentially expressed genes mapped to M1 SF370 genome. Fold-change ratios of significantly differentially expressed genes (P<0.05) are mapped to ORFs of the M1 SF370 genome (the M1 strain used as core for the microarray). SF370 prophages are shown, including those absent in M1T1. The graph shows sets of contiguous genes with similar coexpression patterns. A higher resolution version of the figure is provided online as Figure S4.

**Figure 4:**
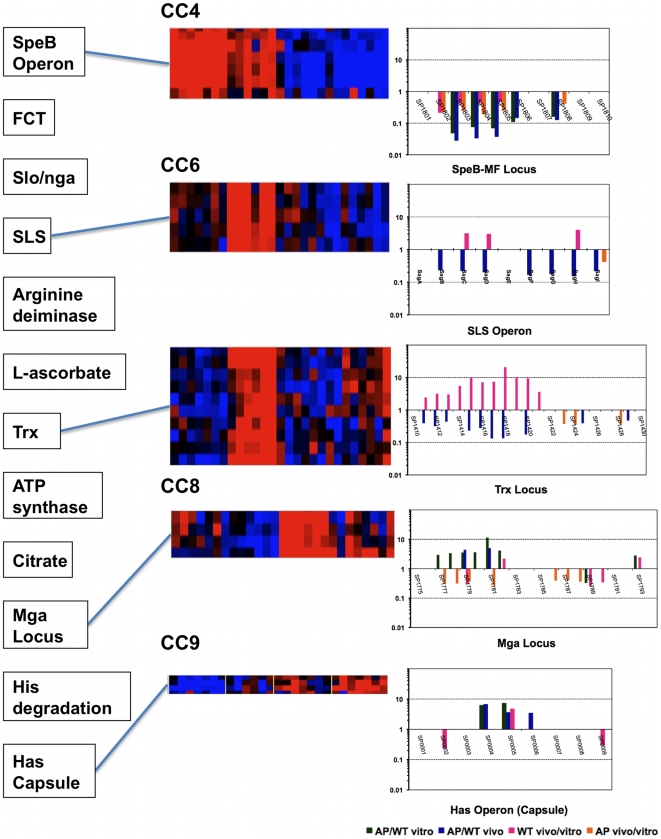
Examples of clusters of biological interest. The correlation between different methods of analysis of five clusters is shown. The left panel includes the different clusters detected by the NC method. The middle panels display heat maps of five coexpression clusters representing different patterns (CC4, SpeB operon; CC6, SLS operon and Trx locus; CC8, Mga locus; CC9, Has operon). The corresponding NC graphs for genes within these clusters are shown in the right panels.

Inspired by the recently described neighbor clustering method for microarray analysis [41], we also mapped the significant (P<0.05) differential expression ratios of different genes to their chromosomal loci (Fig. 3 and Fig. S4) to allow the visualization of coexpressed contiguous genes, including operons. This mapping highlighted genes that might otherwise have been overlooked by the CC or expression ratio methods. Examples of neighbor clusters (NCs) enriched in differentially expressed genes are a locus involved in L-ascorbate utilization (SPy0175-SPy0179); the citrate lyase locus (SPy1186-SPy1191); the Trx chromosomal locus (SPy1582-SPy1596) that includes the recently described, CovR-repressed two-component response regulator TrxR [42]; and the well-studied Mga (SPy2010-SPy2025), SpeB (SPy2037-SPy2042), and capsule synthesis (SPy2200-SPy2202) loci (Fig. 3 and Fig. S4). NC analysis also allowed us to visualize genomic clusters that are similarly regulated in vitro and in vivo, and those that are reciprocally regulated. For example, in both the SLS operon and the Trx locus, we observed a striking difference between the effects of the *covS* mutation and the in vivo adaptation (Fig. 4, CC6). Genes of these two chromosomal clusters are significantly downregulated in AP compared to WT bacteria, but significantly upregulated in WT-vivo relative to WT-vitro conditions (Fig. 4, CC6).

As a final step of our multifaceted data analysis approach, we tabulated significant transcriptional changes of individual genes that encode known regulators and well-studied virulence factors (Table 3). Focusing on those genes highlights the pathogenesis-related changes and allows easy comparison of our data with those in the literature [11], [12], [13], [16], [27], [37].

### Data integration reveals genomic subsystems influenced by the *covS* mutation in vitro and in vivo

**Figure 5:**
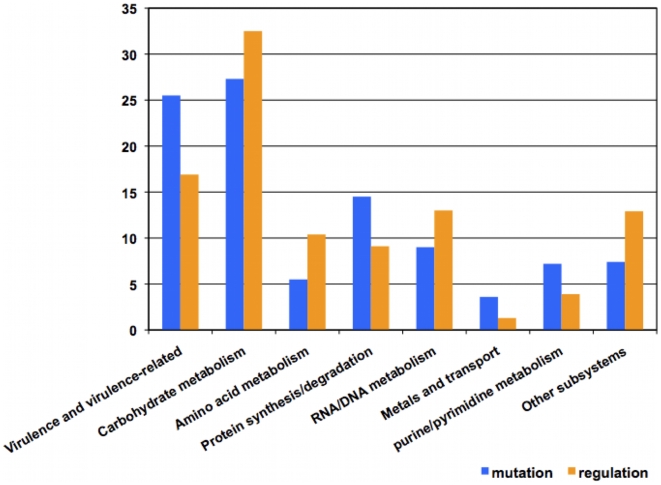
Genomic subsystems represented by the significantly differentially regulated genes. Annotations and subsystem classification are based on NMPDR [79] annotations as of October 2009. Subsystem classification has been manually verified and amended when necessary.

By combining different strategies for microarray data analysis, we took advantage of the strengths of each strategy to generate biologically relevant gene sets rather than gene lists ordered solely according to statistical parameters. The next stage in our analysis was to integrate microarray data, moving from the gene/cluster level to the level of biological subsystems. A subsystem is a part of a genome that represents a functional module, e.g., an operon, a cellular pathway, a regulon, or a complex regulatory network [43]. The genes perturbed by the different experimental conditions of this study correspond to multiple subsystems (Fig. 5). Of interest, among the genes of known function, 25% of those whose transcription was modulated as a consequence of the *covS* mutation are virulence-related and another 25% are related to carbohydrate metabolism pathways, which are connected to virulence in streptococci [44], [45], [46].

When we attempted to correlate the array results with known GAS regulons, some differentially expressed gene sets could be fitted into previously described regulatory networks while others did not belong to any such networks. For example, the upregulation of Ska, SLO/Nga, and the Has operon is a pattern that reflects the inhibition of CovR repression, whereas the downregulation of both SpeB and SLS operons is a pattern associated with the transcriptional regulator RopB/Rgg [47], [48] but that may also reflect augmented CovR repression [20].

Although CovS is expected to modulate many known CovR-repressed subsystems, certain operons may be differentially modulated or may be influenced by other regulator(s). Alternatively, the mutation in *covS* may have different effects on CovR-mediated regulation of different genes [26]. In fact, our in vivo data show that *covR* itself is downregulated in vivo in both WT and AP strains (Fig. 2, CC2 and Table S1); however, the SLS operon is upregulated in vivo in WT bacteria, suggesting that its regulation is not solely modulated by CovRS. It is possible that SLS is regulated by the catabolite control protein (CcpA), which was recently shown to be another key transcriptional regulator of the SLS operon [46] and which may override CovR-mediated repression of SLS. Another complex regulatory pattern is exhibited by the genes of the Trx locus, which include a two-component regulatory system, TrxRS. The TrxRS system reportedly responds to a yet-to-be-identified extracellular signal while TrxR itself is directly regulated by CovR [42]. Most genes within the Trx locus are downregulated in AP GAS relative to WT, but upregulated in the WT bacteria in vivo (Fig. 2, CC6 and Fig. 3) suggesting the possibility of CovS-dependent in vivo signaling.

One last factor that adds to the complexity of data integration analysis and that may explain unexpected transcriptional patterns is that regulators are often controlled by sensitive feedback mechanisms. Inactivation or downregulation of one regulator may eventually perturb the entire system, and several other regulators are likely to become engaged in compensatory mechanisms to maintain cellular homeostasis.

## Discussion

### Group A streptococcal sociomicrobiology

It is now well established that bacterial populations often consist of heterogeneous communities rather than genetically identical cells with synchronized gene expression profiles [49], [50], [51]. In this study, we show how gross changes in the bacterial environment, such as the onset of infection, can profoundly perturb the sociomicrobiological structure of the bacterial population, driving a minor subpopulation with a mutant hypervirulent phenotype to thrive, prevail, and cause severe disease. Although a number of informative studies have compared gene and protein expression in virulent and hypervirulent GAS isolates [11], [12], [13], [16], [26], [27], [37], none have captured the transitional state that reflects the dynamics of the bacterial struggle to survive in a new host environment. Normally, capturing such evolutionary events in real time is difficult, both because multiple sampling of the bacteria in vivo is often unfeasible due to their dissemination, and because the fittest members of the community usually overtake the rest of the population rather rapidly, thereby hampering the ability to capture dynamic changes associated with the restructuring of the bacterial community at different host niches. Our subcutaneous chamber model of infection allowed us to sample the inoculated WT and AP bacteria at specific times post-infection, during their adaptation to their new environment, to determine how their interaction with the host affects their community structure and their transcriptional reprogramming. In doing so, we were able to dissect changes in gene expression associated with niche adaptation from those resulting from restructuring of the bacterial community, where undetected minority members in vitro became the new majority in vivo, armed with the necessary tools to survive in their new niche. These in vivo-selected bacteria with the AP phenotype could be differentiated from the WT bacteria by mutations that are clustered in the sensor kinase-encoding *covS* gene in AP bacteria [14], [27]. In this study, we captured dynamic events underlying the phenotypic switch resulting from this population restructuring.

### Environmental adaptation vs. *covS* mutation

In vitro, the growth of these two variants of the M1T1 bacteria was comparable and, although 137 (5.9%) of their genes were differentially expressed (Table 2), in vitro phenotypic screening revealed negligible differences (Table 1). This suggests that the majority of in vitro vs. in vivo differentially expressed genes between the WT and AP variants may be involved in niche adaptation. This finding is in agreement with studies showing that the CovRS system is mainly linked to the regulation of virulence factors and virulence-associated pathways [19], [20], [24], [26], [27], [28], [52], [53], [54], [55].

Indeed, several virulence genes were among the 137 in vitro differentially expressed genes between these two M1T1 variants. Upregulated gene sets in AP vs. WT bacteria in vitro included Mga locus genes (e.g., *sic*, *emm*, *scp*); hyaluronic acid capsule-encoding genes (*hasABC*); and genes encoding the toxins SpeA, SclA, SLO, and the streptodornase Sda1 (Fig. 2, Fig. S2, and Table 3). Other well-studied gene sets were downregulated in AP bacteria grown in vitro. Besides metabolic gene sets involved in trehalose and ascorbate utilization, several known virulence factors were downregulated in AP bacteria, and these included the SpeB operon, GRAB, EndoS, and CAMP factor. SpeB is a major virulence factor in GAS pathogenesis and its expression is regulated by different systems [40], [56], [57], [58], including the CovRS system [20], [53]. Whereas SpeB expression may be important in the initial stages of skin infections [29], [35], its downregulation has been associated with an invasive and hypervirulent phenotype [13], [14], [59]. The downregulation of GRAB makes biological sense because this protein binds alpha-2-macroglobulin in blood [60] to protect the bacteria against its own protease, SpeB, and thus the bacteria no longer need to express high levels of GRAB when SpeB is not expressed. Similarly, EndoS, an IgG protease, and CAMP, a hemolysin, are likely needed in blood but with no defined function in subcutaneous or deep tissue. Their downregulation in AP is thus consistent with the hypothesis that this hypervirulent variant is adapted for deep infection. In addition, the downregulation of SpeB, EndoS, and CAMP is in agreement with our previous proteomic results [12] as well as other published transcriptional analyses [13], [27].

As both bacterial variants were subjected to the host environment, they underwent additional changes in gene expression. In vivo, both WT and AP bacteria downregulated a substantial number of their genes (127 genes in CC1 and CC2, Fig. 2 and Table S1), and upregulated many fewer (24 genes in CC3, Fig. 2 and Table S1). By parsing these differentially expressed genes into operons, subsystems, and functional pathways, we found that many reflect the engagement of several regulatory networks involved in metabolic adaptation and immune camouflage or evasion of host defenses. Many of the in vivo changes that both the WT and AP bacteria underwent are suggestive of major metabolic reprogramming associated with the transition from a saprophytic lifestyle in the carbohydrate-rich laboratory culture medium to a parasitic lifestyle in the vascularized and the anaerobic subcutaneous environments rich in peptides, amino acids, nucleotides, and different types of complex carbohydrates. For example, among the downregulated gene sets are those involved in citrate metabolism, arginine degradation, and de novo pyrimidine synthesis; many of those gene sets were previously reported to be perturbed upon blood inoculation and to be controlled by CovR [61] and RopB/Rgg [38].

We also found that many ribosomal and cell-division proteins were downregulated in vivo, which suggests that the cells may be slowing down protein synthesis to preserve energy, or redirect this energy to colonizing the host and evading its immune system. The downregulation of arginine deiminase, a streptococcal immunogen and a potential vaccine target (Henningham et al., submitted), is also suggestive of immune evasion. Among the upregulated genes are dipepetide and sucrose-specific transporters, whose upregulation supports the hypothesis that the bacteria are switching diets [39] and attempting to scavenge nutrients available in their new host environment.

Several phage genes are split between the upregulated and downregulated gene clusters; this may indicate a stress-dependent reprogramming of prophage induction and gene expression that needs to be explored in future studies.

Interestingly, what this study revealed is that some of the downstream regulatory effects of the *covS* mutation in AP bacteria are only manifested in vivo. For example, several gene sets (e.g., SLS genes, Trx-locus genes, L-ascorbate utilization genes) were upregulated in WT bacteria in vivo, but were mostly silenced in AP bacteria in vivo (Fig. 2, CC6). On the other hand, genes encoding the fibrinolytic enzyme streptokinase, Ska, a RofA-like transcriptional regulator, several cell division proteins, and some phage proteins were only upregulated when the AP bacteria sensed the in vivo environment (AP-vivo, Fig. 2, CC10). Both these expression patterns suggest CovS-dependent in vivo signaling via different downstream pathways.

Finally, genes encoding the antiphagocytic M protein, and the toxins streptolysin O and NADGH were among few genes that were upregulated in *all* conditions except WT-vitro (Fig. 2, CC7), which suggests that their transcription is dependent on multiple signals, including signals from the host as well as *covS*-dependent cues. Taken together, these results demonstrate how the murine model has allowed us to finely dissect two classes of events affecting GAS sociomicrobiology: those related to reversible transcriptional adaptation and those irreversibly caused by the *covS* mutation in AP bacteria.

### Microarray validation and analysis

Although our microarray studies were extensive (28 arrays for 4 conditions), we ran qPCR to validate the microarray findings, focusing on virulence genes that are biomarkers of the bacterial switch to a hypervirulent phenotype. These include SpeA, SpeB, Sda1, M protein, and SIC [data published in 14]. Comparing the microarray data in this study with previously published work provides further validation of these results. For example, the in vitro microarray results are consistent with our previous proteomic studies of the WT and AP secreted bacterial proteomes [12] as well as with other recently published studies of the in vitro transcriptome of closely related strains [13], [26], [27].

Importantly, we did not base our analysis on single measurements of gene expression, nor did we focus on individual genes; rather, we used gene expression data from biological replicates to rule out biological variability (especially between bacteria recovered from different mice) and to look for changes in gene clusters, operons, and pathways. In doing so, we were able to assess trends across experiments rather than absolute numerical values that could vary due to technical rather than biological factors. We believe that this approach provided more confidence in the final assessment of which genes/pathways were expressed similarly or differentially in both WT and AP bacteria in vitro and in vivo. It also allowed us to dissect changes related to niche adaptation from those reflecting the selection of the fittest members of the bacterial community when faced with different environments and conditions.

The use of multiple microarray analysis strategies and the integration of their results strengthened this study since each strategy has advantages and disadvantages. For example, coexpression clustering—one of the earliest developed microarray analysis tools [62], [63]—provides an overall view of expression patterns of different genes, and greatly helps dissect and demonstrate the effect of each condition on the overall transcriptome. However, coexpression analysis alone can miss some biologically relevant genes that could fail the statistical tests for non-biological reasons (e.g., poorly hybridizing probes, low signal-to-noise ratio, or high variance) and may instead include some irrelevant genes in isolation of their biological networks [41]. In bacteria, biochemical pathways, virulence systems, and multimeric proteins are often encoded next to each other by chromosomally contiguous or clustered genes [64], [65]. Analyzing coexpressed genes in the context of chromosomal clusters is thus most informative. Indeed, neighbor clustering [41] has allowed the enrichment for contiguous gene sets and the prediction of more context-related expression patterns. Genes in neighbor clusters could have been otherwise overlooked either because they were misannotated but their co-occurrence in known clusters revealed their importance, or because they did not pass the statistical tests but, since many bacterial transcripts are polycistronic, the expression of two or more members of a polycistron strongly suggests that the whole operon is expressed.

Besides these two clustering methods, expression ratios provided pairwise comparison, thereby allowing the quantification of the impact of each individual change of condition on overall gene expression (Table 2) as well as on specific genes of interest (Table 3). However, the use of ratios alone may be misleading, especially when they are calculated between two transcripts with low expression levels, resulting in spurious ratios of low biological significance. Similarly, the common use of statistical constraints with ratio calculation (e.g., two-fold ratios and P values <0.05) filters out many biologically relevant genes that are true positives. Finally, the use of operons, subsystems, and pathways to describe the array results avoids inappropriately building conclusions on the transcriptional changes of individual genes and thus better reflects biologically relevant perturbations in specific pathways.

### The bigger picture: niche adaptation and the evolution of hypervirulence in *S. pyogenes*

Having used multiple strategies and integrated the PM data with the in vitro and in vivo microarray data, we propose the following hypotheses about streptococcal niche adaptation and switch to hypervirulence:

(1) Exposure to the in vivo environment perturbs a substantial number of GAS genes and regulatory networks. This has been demonstrated by the larger number of genes affected during in vivo adaptation of both M1T1 variants compared to those affected by the *covS* mutation (Table 2). However, many of the genes modulated in vivo seem to be involved in metabolic reprogramming and stress responses rather than pathoadaptation and virulence. This is why we believe that transcriptional reprogramming by environmental adaptation alone was insufficient to provide WT bacteria with an in vivo survival advantage. In fact, WT bacteria underwent the most dramatic in vivo transcriptional reprogramming (Table 2); yet, they failed to survive since after five to seven days post-infection, they became extinct, and only AP bacteria could be isolated from the mice chambers [16].

(2) The *covS* mutation in AP bacteria seems to be preferentially modulating virulence mechanisms, as about 25% of the known genes perturbed by the mutation belong to virulence subsystems (Fig. 5) and many of the other perturbed genes are indirectly associated with virulence (e.g., carbohydrate metabolism [66] and arginine utilization proteins [38]). In addition, the pathoadaptive clusters (CC4-CC10 in Fig. 2) are more enriched in virulence-related genes than the clusters involved in in vivo adaption (CC1-CC3 in Fig. 2). This finding suggests that AP bacteria are somehow “pre-adapted” to invasiveness and, consequently, when injected into mice, they do not undergo much virulence-related changes as they already possess a thicker capsule, lack a functional SpeB, are equipped with surface virulence proteins, and secrete ready-to-use toxins, including the potent DNase, Sda1. However, the degree of AP invasiveness might vary depending on the route of infection and animal model [15].

It is noteworthy that SpeB expression has a dominant effect, since even if secreted in low amounts by a minor subpopulation, this broad-spectrum protease would still be able to degrade, fully or partially, many GAS virulence proteins, including Sda1, thereby rendering the bacteria vulnerable to different effector mechanisms of the host's innate immune system including neutrophil killing [14], [67], [68]. Thus, a complete shutdown of proteolytically active SpeB is essential to preserve effective extracellular virulence factors [15]. This dominant SpeB effect may explain why WT bacteria parish in vivo even though they partially downregulate SpeB transcription (Table 3).

(3) The GAS genome has at least 13 two-component regulatory systems [69] in addition to several stand-alone transcriptional regulators [47], [70]. However, the CovRS system is a major player, among these regulators, in driving the bacterial adaptation to the host's environment and regulating virulence directly or through other downstream regulators [20], [54]. It is thus counterintuitive that bacteria lacking the important environmental sensor, CovS, would prevail in one of the most stressful environments. However, losing this sensor might be the last resort for these bacteria stranded away from their primary niche and surrounded by hostile immune cells and proteins. From an evolutionary point of view, it is possible to speculate that the bacteria lose their danger sensor to keep their “weapons” constitutively expressed and fight to survive when escape is not an option. Such mutation is likely detrimental to the bacterial long-term survival and dissemination via colonization of new hosts [71], as they need a WT sensor for better adaptability [72] (e.g., in less hostile niches like throat or saliva [26]). An intact CovRS system would offer the bacteria enough versatility to turn on and off many regulatory networks through CovS signaling. This flexible mechanism allows the bacteria to initially hide from the immune system through SpeB-driven camouflaging, i.e. degradation of most of their immunogenic virulence factors [2], [3].

(4) We also show that the impact of the *covS* mutation goes beyond the defined CovR regulon. This finding is in accordance with previous observations that CovR and CovS are not committed to each other, as CovR could be phosphorylated in the absence of a functional CovS [22] and as different *covS* mutations, albeit clustered in the histidine kinase domain, might have different transcriptional effects [27], including opposite effects on different members of the CovR regulon [26].

### Concluding remarks

In conclusion, we have established a model that allowed us to resolve two sets of complex transcriptional events: (i) those occurring in response to the host environment and (ii) those caused by a mutation in *covS* sensor kinase. We believe that our results offer a proof of principle that in vivo-extracted RNA can provide transcriptional profiles that better reflect the complexity of heterogeneous bacterial communities, and, as shown in this study, can provide a transcriptional snapshot of a bacterial population right before its extinction. The use of in vivo-driven RNA in understanding virulence has been appreciated in streptococcal research [73], but the technique has not previously been used to explain the switch to hypervirulent, invasive phenotypes or to dissect heterogeneous microbial subpopulations.

This study is a first step towards exploring the sociomicrobiology of invasive GAS in vivo. Having captured snapshots of different transcriptional programs within the same bacterial community, we plan to follow with single cell studies [74], [75] of bacteria associated with immune cells to further dissect the different roles played by members of the same bacterial community.

## Materials and Methods

### Ethics statement

All animal experiments were conducted according to the Guidelines for the Care and Use of Laboratory Animals of the National Institutes of Health and approved by the institutional animal care and use committees at the University of Cincinnati, OH, USA and the VA Medical Center, Memphis, TN and Cincinnati, OH, USA.

### Bacteria and culture media

We used the clinical isolate, GAS 5448 [4], [59], representative of the clonal M1T1 strain [2], [10] as well as its animal-passaged (AP) descendant 5448 AP [12], which was shown to be a natural *covS* mutant [14]. Bacteria were grown in vitro in THY medium (Todd Hewitt broth, DIFCO, Detroit, MI, supplied with 1.5% yeast extract, DIFCO). Cultures were routinely tested for purity on blood agar plates (Becton Dickenson, Franklin Lakes, NJ) and for proteolytic activity on casein-Columbia agar plates as described previously [14], [48].

### Biolog Phenotype Microarray experiments and analysis

Both bacterial strains GAS 5448 WT and 5448 AP were analyzed by the Biolog® Phenotype Microarray (PM) technology. The analysis and data processing were performed by the Biolog® team (Hayward, CA). Duplicate arrays were run, and the average of the two runs was calculated. Only results that were significantly different in *both* runs are reported as significant.

### Expression microarrays

Oligomers (70-mers) of the M1-based microarrays were obtained from Dr. Kevin McIver and Dr. June Scott, and printed in the Molecular Resource Center, UTHSC by the use of MicroGrid II (Genomic Solutions, Ann Arbor, MI). Each array consists of 2,346 oligomers that represent all open reading frames (ORFs) in M1 GAS strain SF370 (GenBank accession # NC_002737 [76]) in addition to ORFs from prophages in strains MGAS8232 (GenBank accession# NC_003485 [77]) and MGAS315 (GenBank accession # NC_004070 [78]). These oligomers were printed in duplicates at different locations of polyamine-coated glass slides (Telechem International Inc., Sunnyvale, CA) together with so-called alien DNA (Stratagene, La Jolla, CA) negative controls, which are synthetic DNA sequences with no homology to any DNA in current sequence databases.

### RNA extraction

To obtain high-quality RNA from GAS cells, we followed a multi-step protocol. First, we mixed the bacteria with a Lysing Matrix B (QBiogene, Irvine, CA) and used a FastPrep instrument (QBiogene) to shear the bacterial walls. Then, we extracted total RNA from the sheared bacteria using RNeasy kits (Qiagen, Valencia, CA), treated it with DNase Turbo (Ambion, Austin, TX) for 1 h to remove contaminating genomic DNA, and further purified the samples using RNeasy columns (Qiagen). Sometimes we used RNeasy MinElute columns to concentrate RNA when the yield was low, typically in case of in vivo-recovered RNA. We confirmed the absence of genomic DNA in the samples by running 40-cycle PCR reactions using GAS *speB* or gyrase primers as described elsewhere [12], [27].

### Animal model

For in vivo experiments, we used the subcutaneous murine Teflon chamber model developed in our laboratory and described earlier [16]. Sterile Teflon chambers were surgically inserted under the skin of age-matched female BALB/c mice. Three weeks after surgery, mice were screened, and only those with sealed subcutaneous chambers containing sterile tissue chamber fluid (TCF) were selected for the experiments. The bacterial inoculum was prepared as follows: bacteria were grown overnight in THY medium then subcultured again for 18 h, washed twice in sterile phosphate-buffered saline (DIFCO).

To recover enough RNA for downstream experiments, we inoculated the mouse chambers with 2×10^8^ CFU/mouse (100 ul of a 2×10^9^ CFU/ml culture). After 24 h, we used the bulk of the recovered bacteria for immediate RNA extraction (as detailed above) with no additional culturing, and kept a small aliquot intact to verify retention of SpeB phenotype using protease screens on casein-agar plates. RNA from bacteria homogeneously expressing or not expressing proteolytic activity was used for transcriptome profiling while RNA from the few populations that exhibited mixed SpeB^+^ and SpeB^−^ phenotypes was excluded.

### Preparation of labeled cDNA probes and microarray hybridization

To convert the bacterial RNA to labeled cDNA ready for hybridization, we used the 3DNA Array 900TM kits (Genisphere, Hatfield, PA; http://www.genisphere.com/array_detection_900.html), which use the dendrimer technology to amplify the fluorescent signal of cDNA. Following the manufacturer's protocol, we tagged each sample with the proprietary capture reagent, mixed equal amounts from both tagged samples (WT or AP, grown in vitro or in vivo), and used the mixed samples to hybridize with the probes on the microarray slides overnight. After the first hybridization, we washed the slides, incubated them with equal amounts of the fluorescent dyes (Alexa Fluor 546 and Alexa Fluor 647, from Genisphere) for 3 h, washed them again, and immediately scanned them. Alternatively, we used DyeSaver (Genisphere) to coat the array slides and protect the dyes from fading when immediate scanning was not possible.

### Design of microarray experiments

In planning the microarray experiments, we chose to follow a cyclic design according to which we compared, for example, WT-vitro to AP-vitro, then AP-vitro to AP-vivo, then AP-vivo to WT-vivo, and then WT-vivo to WT-vitro (Fig. 1). This scheme allowed an all-to-all comparison without the need for duplicate arrays for each pair of conditions. Because we used a two-color hybridization approach, this scheme also controlled for non-specific hybridization, since each comparison was repeated at least twice with the fluorescent dyes flipped. At least three biological replicates (i.e., samples recovered from three different mice or three different in vitro cultures) of each condition were tested, and each biological replicate was run at least twice. In addition, the probes were already printed in duplicates on the glass slides. This conservative design minimizes biological variability caused by mouse-to-mouse or culture-to-culture variations and reveals differences due only to dynamic changes in population structure or gene regulation. The possibility that some true positive results may have been missed because of this design was compensated by studies assessing the expression of multiple genes in the same operons or chromosomal clusters as detailed in the Results section.

### Analysis and annotation of microarray data

We scanned the arrays using GenePix 4000B scanner (Axon Instruments/Molecular Devices, Sunnyvale, CA) and we performed the primary analysis using the GenePixPro 4.0 software (Axon Instruments/Molecular Devices) The primary analysis included spot finding, alignment and adjustment, fluorescence normalization, flagging out poorly hybridized spot, and background subtraction. We performed subsequent analyses using multiple tools, including Microsoft Excel, GeneSpring (Agilent Technologies, Santa Clara, CA), as well as custom-written Perl scripts that are integrated in the NMPDR (http://www.nmpdr.org) [79] and SEED [43] platforms; these scripts allowed calculation of mean fluorescence values and ratios, filtration of low signal-to-background ratios, clustering and sorting results from different arrays, and finally uploading the results to the SEED website (http://seed-viewer.theseed.org) to allow the visualization of array results. Additional clustering, statistical analysis, and generation of gene lists and Venn diagrams were performed by multiple tools in the GeneSpring suite (Agilent Technologies). All genome annotations and subsystems data used in this study were obtained from the NMPDR database [79]. All raw microarray data were submitted to the NCBI Gene Expression Omnibus (GEO) in accordance with MIAME standards (GEO accession numbers: GEO platform GPL9701 and series GSE19103: samples GSM473346 through GSM473374). Moreover, all raw data as well as GeneSpring analysis folders are made available online (http://host-pathogen.net/publications/Aziz_2010_Arrays/microarrays).

## Supporting Information

Figure S1 Biolog PM consensus results. A consensus Phenotype Microarray (PM) analysis chart generated from two sets of 20 plates that were run twice for each the wild type (WT) and animal-passaged (AP) bacteria over a 48 h time period. Each well of each plate represents a different condition (nutrient source, antimicrobial, pH or salt concentration, etc.). The red curves represent AP and the green ones represent WT growth curves. Yellow color is the result of superimposition of red and green areas.
(6.26 MB TIF)

Figure S2 A higher resolution version of Fig. 2. Because Fig. 2 dimensions are hard to fit in the print paper size, this larger online version may help readers see the details.
(25.80 MB TIF)

Figure S3 Microarray results summary statistics.
(3.60 MB TIF)

Figure S4 A higher resolution version of Fig. 3. Because Fig. 3 dimensions are hard to fit in the print paper size, this larger online version may help readers see the details.
(0.70 MB PNG)

Table S1 All array data used to generate the pathovivogram (Figs. 2 and S2).
(0.30 MB XLS)

Table S2 Gene lists reflecting significant (P<0.05) differential expression ratios between each pair of conditions.
(0.28 MB XLS)
