# Supplementary figures and images for: Microevolution of Group A Streptococci In Vivo: Capturing Regulatory Networks Engaged in Sociomicrobiology, Niche Adaptation, and Hypervirulence
Source: PLoS One. 2010 Apr 14;5(4):e9798. doi: 10.1371/journal.pone.0009798 (PMC2854683; doi:10.1371/journal.pone.0009798)

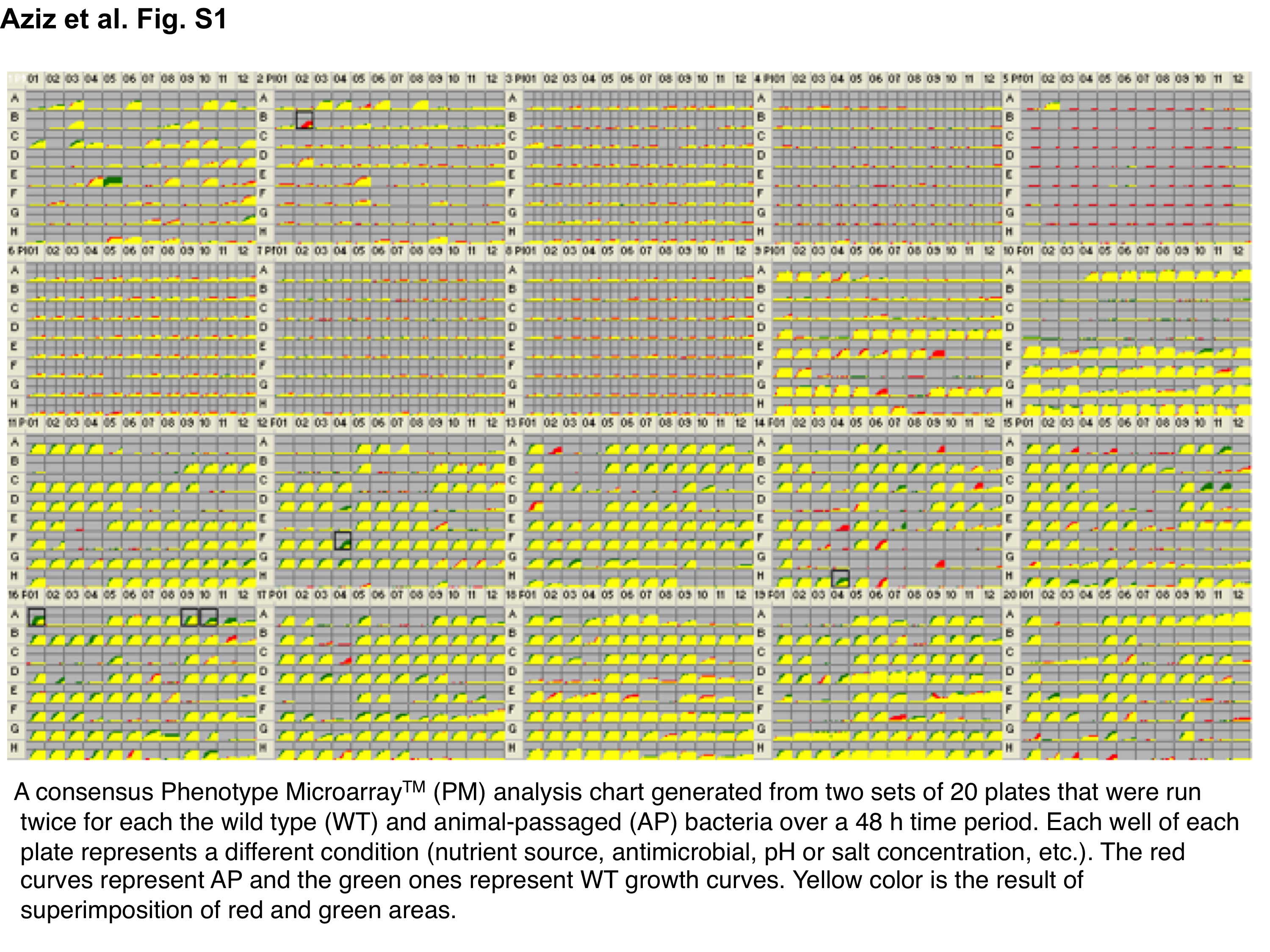

Supplement: Figure S1 — Biolog PM consensus results. A consensus Phenotype Microarray (PM) analysis chart generated from two sets of 20 plates that were run twice for each the wild type (WT) and animal-passaged (AP) bacteria over a 48 h time period. Each well of each plate represents a different condition (nutrient source, antimicrobial, pH or salt concentration, etc.). The red curves represent AP and the green ones represent WT growth curves. Yellow color is the result of superimposition of red and green areas. (6.26 MB TIF) [file pone.0009798.s001.tif]

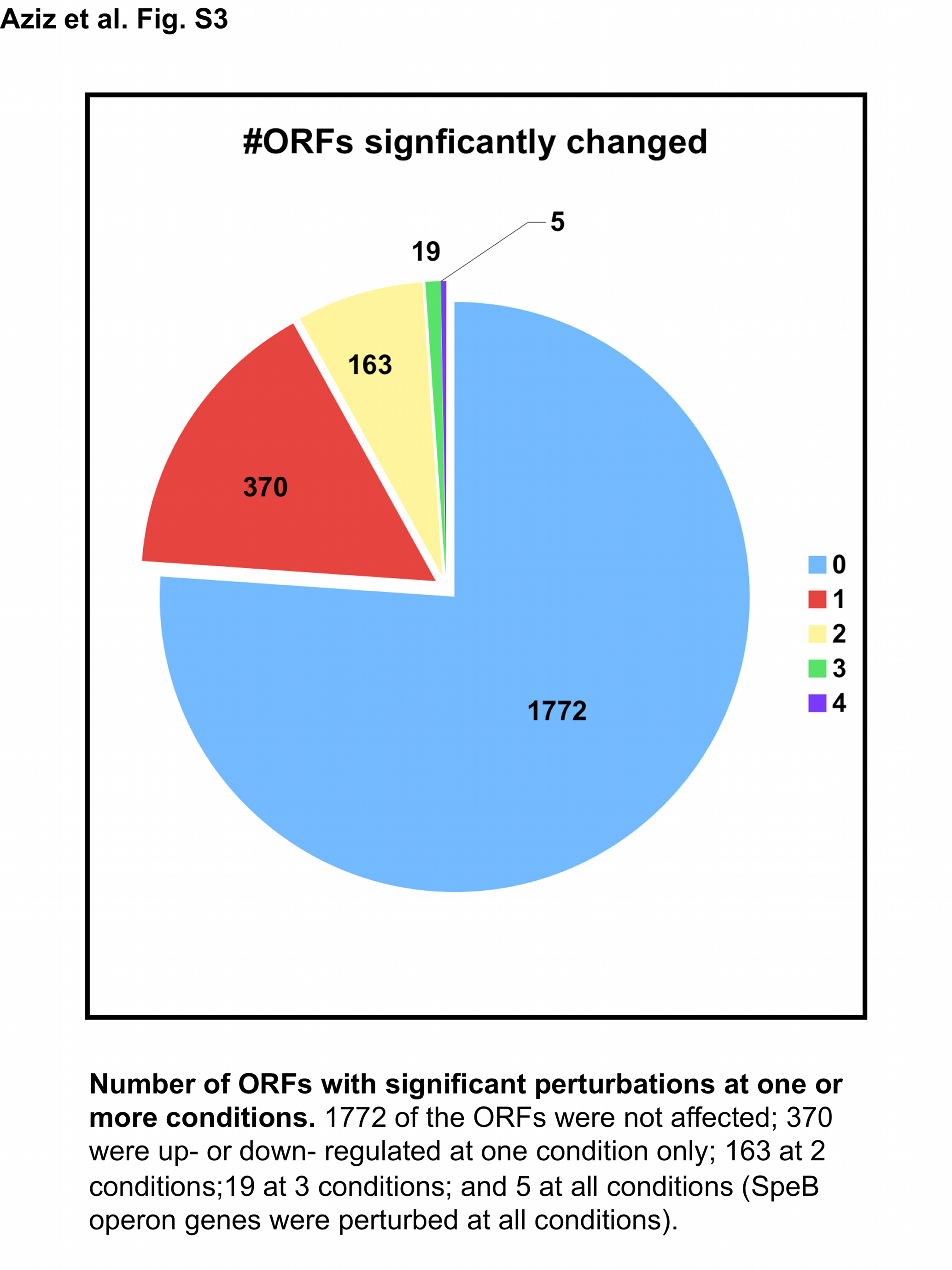

Supplement: Figure S3 — Microarray results summary statistics. (3.60 MB TIF) [file pone.0009798.s003.tif]

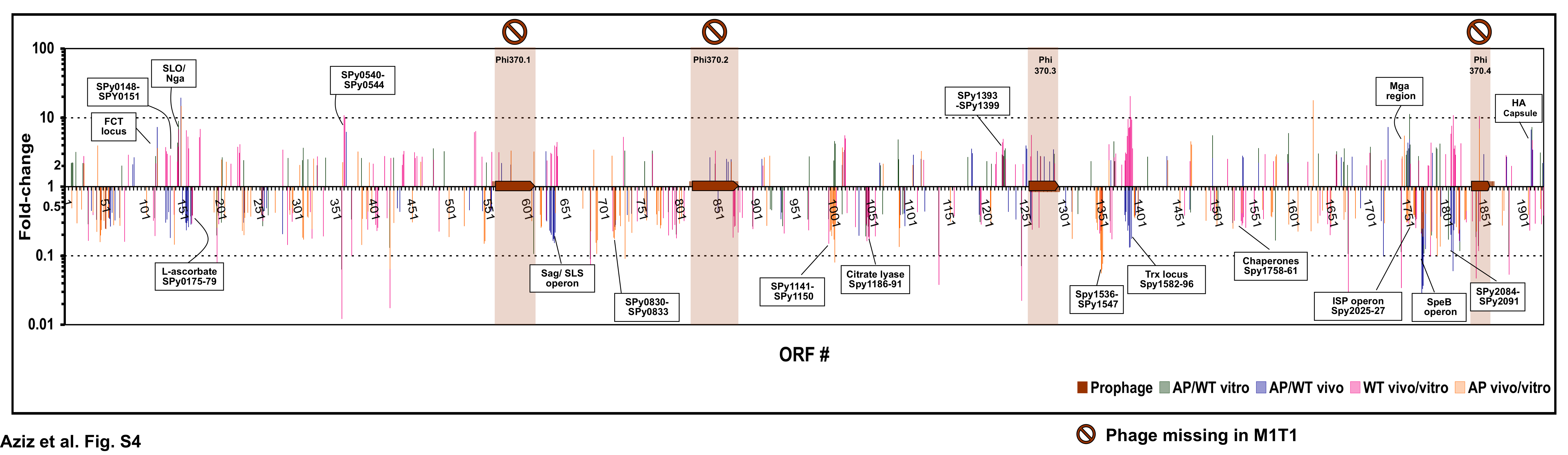

Supplement: Figure S4 — A higher resolution version of Fig. 3. Because Fig. 3 dimensions are hard to fit in the print paper size, this larger online version may help readers see the details. (0.70 MB PNG) [file pone.0009798.s004.png]
